# A Review on Occurrence and Spread of Antibiotic Resistance in Wastewaters and in Wastewater Treatment Plants: Mechanisms and Perspectives

**DOI:** 10.3389/fmicb.2021.717809

**Published:** 2021-10-11

**Authors:** Cansu Uluseker, Krista Michelle Kaster, Kristian Thorsen, Daniel Basiry, Sutha Shobana, Monika Jain, Gopalakrishnan Kumar, Roald Kommedal, Ilke Pala-Ozkok

**Affiliations:** ^1^Department of Chemistry, Bioscience and Environmental Engineering, Faculty of Science and Technology, University of Stavanger, Stavanger, Norway; ^2^Department of Electrical Engineering and Computer Science, Faculty of Science and Technology, University of Stavanger, Stavanger, Norway; ^3^Department of Chemistry and Research Centre, Aditanar College of Arts and Science, Tiruchendur, India; ^4^Department of Natural Resource Management, College of Forestry, Banda University of Agricultural and Technology, Banda, India

**Keywords:** antibiotics, antibiotic resistance genes, antibiotic resistant bacteria, spread mechanisms, wastewater treatment plants

## Abstract

This paper reviews current knowledge on sources, spread and removal mechanisms of antibiotic resistance genes (ARGs) in microbial communities of wastewaters, treatment plants and downstream recipients. Antibiotic is the most important tool to cure bacterial infections in humans and animals. The over- and misuse of antibiotics have played a major role in the development, spread, and prevalence of antibiotic resistance (AR) in the microbiomes of humans and animals, and microbial ecosystems worldwide. AR can be transferred and spread amongst bacteria *via* intra- and interspecies horizontal gene transfer (HGT). Wastewater treatment plants (WWTPs) receive wastewater containing an enormous variety of pollutants, including antibiotics, and chemicals from different sources. They contain large and diverse communities of microorganisms and provide a favorable environment for the spread and reproduction of AR. Existing WWTPs are not designed to remove micropollutants, antibiotic resistant bacteria (ARB) and ARGs, which therefore remain present in the effluent. Studies have shown that raw and treated wastewaters carry a higher amount of ARB in comparison to surface water, and such reports have led to further studies on more advanced treatment processes. This review summarizes what is known about AR removal efficiencies of different wastewater treatment methods, and it shows the variations among different methods. Results vary, but the trend is that conventional activated sludge treatment, with aerobic and/or anaerobic reactors alone or in series, followed by advanced post treatment methods like UV, ozonation, and oxidation removes considerably more ARGs and ARB than activated sludge treatment alone. In addition to AR levels in treated wastewater, it examines AR levels in biosolids, settled by-product from wastewater treatment, and discusses AR removal efficiency of different biosolids treatment procedures. Finally, it puts forward key-points and suggestions for dealing with and preventing further increase of AR in WWTPs and other aquatic environments, together with a discussion on the use of mathematical models to quantify and simulate the spread of ARGs in WWTPs. Mathematical models already play a role in the analysis and development of WWTPs, but they do not consider AR and challenges remain before models can be used to reliably study the dynamics and reduction of AR in such systems.

## Introduction

Antibiotic substances are by far the most powerful tools available for the treatment of infectious diseases by inhibition of bacterial cell growth. In addition to being used for the treatment of infections in human patients and farm animals, antibiotics are also routinely given to healthy farm animals to promote growth and proactively prevent disease outbreaks. Antibiotic resistance (AR) is the ability of bacteria to overcome and resist exposure to antibiotic substances, this is made possible by the acquisition of antibiotic resistance genes (ARGs) (Davison et al., 2000; Wright, 2010). Extensive use of antibiotics since the successful purification and mass production of penicillin in the middle of the twentieth century until today has led to an increase in antibiotic resistance, compromising the effectiveness of antibiotics (Davies and Davies, 2010).

Antibiotic resistance is a global and challenging issue (Walsh, 2003; Deurenberg and Stobberingh, 2008; Livermore, 2012). The risk it poses needs to be tackled in a context that combines environmental, and human health, which focuses on the mechanisms that drive biological (growth and exchange) and physiochemical (transport and conversion) spread. Considering human health issues like AR in a context that combines human, animal and environmental factors is the essence of the One Health initiative’s perspective, endorsed by the World Health Organisation (WHO) (One Health Initiative, 2020) and AR has large implications for half a dozen of the United Nation’s (UN’s) sustainable development goals (WHO, 2020). Part of the issue is to understand how ARGs spread in different environments [wastewater, wastewater treatment plants (WWTPs), soil, and receiving aquatic eco-system] to prevent the spread existing and the development of new ARGs (Brooks et al., 2008).

For more than 100 years, the Activated Sludge Process has been and still is among the most widespread wastewater treatment technologies used for the removal of key pollutants from municipal wastewater (Stensel and Makinia, 2014; van Loosdrecht and Brdjanovic, 2014). By bacterial uptake and metabolic conversions of organics and nutrients, cellular growth provides for an auto-catalytical removal process which is further enhanced by settling and recirculation of active biomass as originally proposed by Ardern and Lockett (1914). While bacterial densities in wastewater are normally in the range of 10^5^–10^8^ cells per ml (Tchobanoglous et al., 2014), the enhancement of biomass in modern biological WWTPs increases the bacterial density in the bioreactors by 3 orders of magnitude and selection by sedimentation results in dense bacterial aggregates. Additionally, depending on operating conditions and temperature, there can be very high material turnover (up to 90%) and much higher specific heterotrophic growth rates (up to 13.2 day^–1^) in WWTPs bioreactors than in natural water systems. In WWTP bioreactors, microbial diversity and interactions are ubiquitous and frequent (Daims et al., 2006; Nielsen and McMahon, 2014). High abundance, density, diversity, activity, and interactions in the activated sludge bioreactors would suggest an increased rate of gene transfer, including horizontal and vertical exchange of ARG. Mechanisms and rates at which exchange occur in the microbiome of these systems are now under intense study, and resistomes (all ARG’s in a microbial community) of activated sludge systems are currently being mapped (Manaia et al., 2018).

Established effluent standards set the quality of WWTP effluents based on environmental effect parameters such as the chemical and biochemical oxygen demand, the amount of suspended solids, total nitrogen, number of coliform bacteria, etc. (Directive 91/271/Eec, 1991). It is not known how, or whether at all, these parameters indicate the prevalence of antibiotic resistant bacteria (ARB) and ARGs. Lately, more attention has been paid to examine detection and elimination techniques for ARB and ARGs, in addition to removal techniques for other micropollutants like detergents, pesticides, pharmaceuticals, and personal care products (Łuczkiewicz et al., 2010; Luo et al., 2014). Some WWTPs use extra disinfection units at the end of the biological treatment process, which include chlorination, UV radiation and ozonation, or quaternary advanced treatment techniques such as advanced oxidation processes (AOPs) or membrane filtration. Such unit processes can as this review will show reduce the number of ARB and possibly also ARGs in the WWTP effluent but are costly to operate and may not be as effective as observed in laboratory studies (Auerbach et al., 2007; Zhang et al., 2015, 2016a; Zhuang et al., 2015). Biological removal of organic material from wastewater is linked to the fast growth of microorganisms in the WWTP, and since the biomass builds up some is continuously discarded as excess biological sludge. ARB and ARGs that are present in the biomass of the reactor will also be present in the sludge, therefore further treatment processes of excess sludge need to be considered. We will in the last part of section four of this review go through the current knowledge of how effective the different sludge treatment methods are able in reducing ARB and ARGs.

This review will present the major groups of antibiotics, the major groups of mechanisms for antibiotic resistance in bacteria, and the general bacterial mechanisms for genetic exchange, but only briefly as other reviews already have covered this in general (Wright, 2010, 2011; Pazda et al., 2019; Zhu et al., 2021) and in the context of wastewater treatment. More space is instead given to go through what is known about which wastewater sources show the high occurrence of antibiotic resistance, how antibiotic resistance persists and spreads through WWTPs, and what contributes to this persistence. Previous works in the literature already focused on complete lists of every specific type of ARGs that have been found in WWTPs (Pazda et al., 2019), and on the strength and weaknesses of different methods used to measure and analyze ARB and ARGs content in wastewater (Manaia et al., 2018). Therefore, this review will focus on documenting what is known about the removal efficiency of different treatment processes or technologies, i.e., what are the reported elimination efficiencies of ARGs and ARB for different treatment technologies for both wastewater and sludge, and whether the reported results are consistent. Moreover, in this work special efforts have been put into gathering and reviewing results from studies of elimination of ARGs and ARB in different sludge and biosolids treatment processes, as this has been more or less overlooked in other reviews (Barancheshme and Munir, 2018; Pazda et al., 2019; Bairán et al., 2020; Zhu et al., 2021).

In essence, this systematic review aims to describe the factors that affect the persistence and spread of antibiotic resistance in wastewater treatment and to evaluate current and emerging treatment technologies. For completeness this review documents removal efficiencies for antibiotic substances for different treatment technologies, but it does not aim to discuss the pathways and mechanisms for the breakdown of these substances at length, which has been addressed in a recent review by Zhu et al. (2021). Additionally, this review will also discuss how mathematical models can be used to better understand the dynamics of antibiotic resistance spread in WWTPs. It has been suggested that mathematical modeling can help to quantify and simulate the spread of ARGs in WWTPs, but as this review will show only a few models have been proposed and even fewer have been sufficiently parameterized and validated. It will discuss why, and which challenges remain to be tackled before mathematical models can be used to their full potential. Finally, this review concludes with future directions and some key points that should be prioritized for improving the current state of antibiotic resistance in WWTPs.

## Antibiotic Resistance: Mechanisms, Sources, and Transfer

**FIGURE 1:**
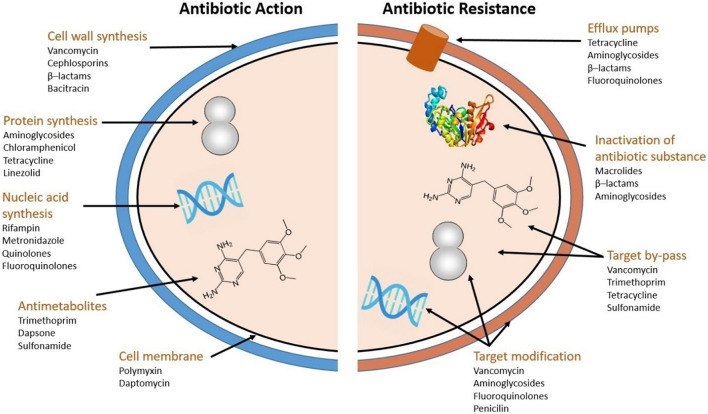
Antibiotic action and resistance mechanisms (adapted from Wright, 2010).

Antibiotics are classified into five major groups, according to their mode of action (Figure 1): (i) Cell wall synthesis inhibition (*vancomycin*, *cephlosporins*, *β–lactams*, *bacitracin*); (ii) Protein synthesis inhibition (*aminoglycosides*, *chloramphenicol*, *tetracycline*, *linezolid*); (iii) Nucleic acid synthesis inhibition (*rifampin*, *metronidazole*, *quinolones*, *fluoroquinolones*); (iv) Antimetabolites (*trimethoprim*, *dapsone*, *sulphonamide*) and; (v) Cell membrane disintegration (*polymyxin*, *daptomycin*). Note that some sources use a coarser division into only four groups (Calderón and Sabundayo, 2007; Kapoor et al., 2017) whereas other use a bit finer division into six (Wanger et al., 2017).

Bacteria have developed four main types of resistance mechanisms against antibiotics (Figure 1; Zhang et al., 2009; Wright, 2010): (i) Efflux pumps, which effectively excrete antibiotics from the cell (Wright, 2011). There are five efflux protein families: ATP-binding cassette (ABC), multidrug and toxic compound extrusion (MATE), major facilitators (MFs), resistance nodulation cell division (RND), and small multidrug resistance (SMR) (Nishino and Yamaguchi, 2001). (ii) Inactivation of antibiotics occurs when the activity of the antibiotic substance is directly hindered by hydrolysis, or by conversion of functional groups etc. (Wright, 2005; Diaz et al., 2014). (iii) Target by-pass: strategies for target by-pass includes creating new pathways to circumvent the originally targeted enzyme, overproduction of the target compound (Munita and Arias, 2016), structural changes in the cell wall (Vila et al., 2007), and prevention of the antibiotic to bind to its target (Wright, 2010). (iv) Target modification: occurs through modification of the antibiotic targets themselves (Wright, 2010). Multiple types of resistance mechanisms may simultaneously confer resistance against the same family of antibiotics (de Sousa Oliveira et al., 2016). Conversely, one type of resistance mechanism can also confer resistance against more than one type of antibiotics.

Wastewater from hospitals and wastewater and waste from animal husbandry together with runoff from manure amended fields are essential ARB and ARGs sources in aquatic ecosystems (Marti and Balcazar, 2013). Hospital wastewaters have especially been shown to contain many ARGs (Zhang et al., 2009; Rowe et al., 2017). One of the first reports dates back to the early 1970s, Grabow and Prozesky (1973) studied the presence of resistant coliforms in hospital wastewater in Pietermaritzburg in South Africa. They found that 26% of coliform bacteria in hospital wastewater had transferable resistance while only 4% of coliform bacteria in municipal wastewater had transferable resistance (Grabow and Prozesky, 1973). The same trend is seen today. Based on several studies done in Europe and Asia, the total ARGs and ARB concentrations in hospital wastewater were 2–9 orders of magnitude higher than municipal wastewater (Li et al., 2015; Lamba et al., 2017; Hutinel et al., 2019). Rowe et al. (2017) showed that the normalized abundance of ARGs in hospital wastewater samples from the Cambridge University Hospitals was 9-folds greater than in samples collected from the effluent lagoon of the University of Cambridge dairy farm and 34-folds greater than in samples from the River Cam source water, which served as background samples for the environment. Another detailed study of wastewater from three different hospitals in Romania showed the presence of genes encoding for resistance for *tetracyclines*, *aminoglycosides*, *chloramphenicol*, *β-lactams*, *sulphonamides*, *quaternary ammonium*, and *macrolide-lincosamide-streptogramin B* antibiotics with abundance levels in as high ranges as 0.01–0.1 copies per 16S rRNA gene copies measured by qPCR (Szekeres et al., 2017). Moreover, in a recent review by Hassoun-Kheir et al. (2020), 37 studies on the occurrence of AR in hospital wastewaters were examined. The review found that 30 (81%) of the studies reported that hospital wastewater contains higher amounts of AR than community wastewater. Furthermore, in a subset of studies where the impact of hospital wastewater on the dissemination of AR in the environment was considered, 25 out of 32 (78%) studies held that hospital wastewaters had an important role as a source of AR to the environment.

**FIGURE 2:**
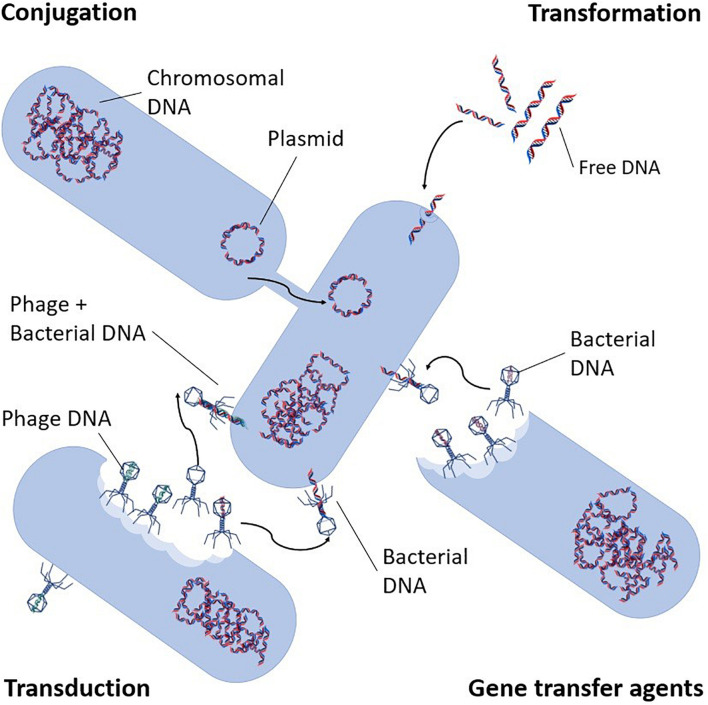
Antibiotic resistance transport mechanisms (adapted from von Wintersdorff et al., 2016).

Apart from the vertical inheritance, antibiotic resistance can be obtained in two ways, through mutation or by horizontal gene transfer (HGT) (Jury et al., 2010). The latter is the most concerning regarding the spread of antibiotic resistance in WWTPs since ARGs can potentially be transferred between organisms effectively and much faster than resistance development through mutations. HGT is a non-reproductive intra- and inter-species transfer of genetic information by means of mobile genetic elements (MGE), such as plasmids and transposons (Barlow, 2009; Huang et al., 2017a). The movement of genes from chromosomes to and between MGEs are mostly facilitated by integrons (Mazel, 2006; Davies and Davies, 2010; Gillings, 2014). There are three different HGT mechanisms for the spread of MGEs. (i) Conjugation: transfer mechanism that requires cell-to-cell contact, where a recipient bacterium acquires genetic material from a donor bacterium, usually in the form of a plasmid (Madigan et al., 2006; Figure 2). (ii) Transformation: intra- and inter-species exchange of genetic information by uptake of, free extracellular suspended DNA, which can only be received by a competent bacterium. Following uptake and translocation to the cytoplasm, it is incorporated into the recipient’s chromosome or into a plasmid (Madigan et al., 2006; Heuer and Smalla, 2007). Finally; (iii) Transduction: involves bacteriophages that transport different types of genetic elements from their host to the receiver (Cano and Colomé, 1988; Snyder and Champness, 2007; Modi et al., 2013), whereupon this is incorporated into the genome of the new host by recombination (Figure 2). There are two types of this mode of transfer, namely generalized and specialized transductions. In generalized transduction, only a segment of bacterial DNA is randomly packed into the bacteriophage head, and bacterial host DNA becomes a part of the DNA of the phage whereas in specialized transduction, both phage and specific bacterial DNA are packed into the head (Chiang et al., 2019). Transduction may also occur *via* gene transfer agents (GTAs), which are DNA carrying structures that resemble bacteriophages, but which do not self-replicate. Although GTAs exact impact has not yet been determined, their potential to act as carriers of resistance in the environment continues their attention (von Wintersdorff et al., 2016).

## Occurrence and Spread of Antibiotic Resistance Genes in Wastewater Treatment Plants

Although antibiotic resistance (AR) occurs naturally at low levels in most ecosystems, the occurrence of ARB and ARGs at high levels is associated with anthropogenic activities. Table 1 shows an overview of resistance genes found in bacteria from wastewater effluents and in aquatic ecosystems. ARGs are frequently detected in WWTPs (Chen and Zhang, 2013; Novo et al., 2013; Rizzo et al., 2013; Manaia, 2014; Table 1), and studies have shown that the ARGs found in wastewaters often reside in clinically relevant pathogenic bacteria (Figueira et al., 2011; Marti et al., 2013a; Hembach et al., 2017). Samples from three different stages of a WWTP in Poland showed that approximately 22, 5, and 9% of *Enterobacteriaceae* strains isolated from (i) the raw sewage in the primary sedimentation tank, (ii) the aeration tank, and (iii) from the effluent, respectively, carried the *intI* integron; and that all strains which carried this integron were resistant to at least three unrelated antibiotics (Mokracka et al., 2012). Note that, although a significant fraction of bacteria in the effluent of this WWTP were still resistant, the above percentages must be interpreted with care as the total number of culturable coliform bacteria in the effluent was reduced with a factor of as much as 10^3^ in the effluent as compared to in the raw sewage (Mokracka et al., 2012). Many of the *Enterobacteriaceae* isolated from a wastewater treatment plant in the study by Amador et al. (2015) were also found to be resistant, and even multi-resistant. The isolates showed resistance against *β-lactam* group antibiotics, including *cefoxitin*, *amoxicillin*, *cefotaxime*, and non *β-lactam* groups antibiotics, including *trimethoprim/sulfamethoxazole*, *ciprofloxacin*, and *tetracycline*. Other studies (Mokracka et al., 2012; Szekeres et al., 2017; Karkman et al., 2018) have also shown that resistance genes against antibiotics, including *tetracycline*, *methicillin* and *sulphonamide* are present in WWTPs. Based on a review of many studies, *tetracycline* (*tet*) resistance genes have been found to be one of the most commonly occurring ARGs in wastewater treatment systems in many countries (Zhang et al., 2009).

**TABLE 1:** Overview of antibiotic resistance genes (ARGs) found in influent, effluent, and activated sludge in wastewater treatment plants (WWTPs) and if their concentrations increase (↑) or decrease (↓) from influent to effluent [adapted from Pazda et al. (2019) and shortened to only include studies that have measured changes in concentration from influent to effluent].

Antibiotics	Antibiotic resistance genes (ARGs)	Sample				Country	References
				
		Influent effluent					
		activated sludge					
*β*–Lactams	*ampR*		+	↓	N.A.	Canada	Biswal et al., 2014
	*bla*_*AmpC*_	+	+	↑	N.A.	Germany	Alexander et al., 2015
	*bla_*CMY*__–__13_*	+	+	↑	N.A.	Sweden	Bengtsson-Palme et al., 2016
	*bla_*CTX*__–__*M*_*	+	+	↓	N.A.	Canada	Neudorf et al., 2017
	*bla_*CTX*__–__*M*_*	+	+	↓	N.A.	Canada	Neudorf et al., 2017
	*bla_*CTX*__–__*M*__–__1_*	+	+	↑	N.A.	Portugal	Amador et al., 2015
	*bla_*CTX*__–__*M*__–__9_*	+	–	↓	+	Poland	Korzeniewska et al., 2013
	*bla_*CTX*__–__*M*__–__12_*	+	+	↑	+	Canada	Biswal et al., 2014
	*bla_*CTX*__–__*M*__–__32_*	+	+	↓	N.A.	Denmark	Laht et al., 2014
	*bla*_*FOX*_	+	+	↑	N.A.	Portugal	Amador et al., 2015
	*bla*_*OXA*_	+	+	↓	+	China	Yang et al., 2014
	*bla*_*OXA*_	+	+	↑	N.A.	Portugal	Amador et al., 2015
	*bla_*OXA*__–__10_*	+	+	↓	+	China	Yang et al., 2014
	*bla_*OX*__*A–46*_*	+	+	↓	+	China	Yang et al., 2014
	*bla_*OX*__*A–58*_*	+	+	↓	N.A.	Denmark	Laht et al., 2014
	*bla_*OX*__*A–58*_*	+	+	↓	N.A.	Finland	Hultman et al., 2018
	*bla_*SHV*__–5_*	+	–	↓	+	Poland	Korzeniewska et al., 2013
	*bla*_*TEM*_	+	+	↑	N.A.	Canada	Biswal et al., 2014
	*bla*_*TEM*_	+	+	↑	N.A.	Portugal	Amador et al., 2015
	*bla*_*TEM*_	+	+	↑	N.A.	Spain	Rodriguez-Mozaz et al., 2015
	*bla_*VIM*__–1_*	+	+	↑	N.A.	Germany	Alexander et al., 2015
	*bla*_*VIM*_-11	+	+	↓	+	China	Yang et al., 2014
Quinolone	*gyrA*	+	+	↓	+	China	Xu et al., 2015
	*parC*	+	+	↓	+	China	Xu et al., 2015
	*gnrC*	+	+	↑	+	China	Xu et al., 2015
	*gnrD*	+	+	↓	+	China	Xu et al., 2015
	*gnrS*	+	+	↑	N.A.	Canada	Neudorf et al., 2017
	*gnrS*	+	+	↑	N.A.	Spain	Rodriguez-Mozaz et al., 2015
	*gnrS1*	+	+	↓	N.A.	Canada	Biswal et al., 2014
Macrolide	*ereA*	+	+	↑	N.A.	Canada	Biswal et al., 2014
	*ereB*	+	+	↑	N.A.	Canada	Biswal et al., 2014
	*ermB*	+	+	↓	+	China	Yang et al., 2014
	*ermB*	+	+	↓	N.A.	Canada	Neudorf et al., 2017
	*ermB*	+	+	↓	N.A.	Germany	Alexander et al., 2015
	*ermB*	+	+	↑	N.A.	Spain	Rodriguez-Mozaz et al., 2015
	*ermF*	+	+	↓	+	China	Yang et al., 2014
	*macB*	+	+	↓	+	China	Yang et al., 2014
	*mef*	+	+	↓	+	China	Yang et al., 2014
	*mph(A)*	+	+	↑	N.A.	Canada	Biswal et al., 2014
Tetracycline	*tetA*	+	+	↓	+	China	Xu et al., 2015
	*tetB*	+	+	↓	N.A.	Canada	Biswal et al., 2014
	*tetB*	+	+	↓	+	China	Xu et al., 2015
	*tetB(P)*	+	+	↑	+	Sweden	Bengtsson-Palme et al., 2016
	*tetC*	+	+	↓	N.A.	Denmark	Laht et al., 2014
	*tetE*	+	+	↑	+	China	Xu et al., 2015
	*tetG*	+	+	↓	+	China	Yang et al., 2014
	*tetM*	+	+	↓	N.A.	Finland	Hultman et al., 2018
	*tetM*	+	+	↓	+	China	Yang et al., 2014
	*tetO*	+	+	↓	+	China	Yang et al., 2014
	*tetQ*	+	+	↓	+	China	Yang et al., 2014
	*tetV*	+	+	↑	+	China	Yang et al., 2014
	*tetW*	+	+	↓	+	China	Yang et al., 2014
	*tetX*	+	+	↓	+	China	Yang et al., 2014
	*tetZ*	+	+	↑	+	China	Xu et al., 2015
	*tet32*	+	+	↓	+	China	Yang et al., 2014
	*dfrA3*	+	+	↑	N.A.	Sweden	Bengtsson-Palme et al., 2016
	*dfrA20*	+	–	↓	N.A.	Canada	Biswal et al., 2014
	*dhfrXV*	+	–	↓	N.A.	Canada	Biswal et al., 2014
	*sulI*	+	+	↓	N.A.	Canada	Neudorf et al., 2017
	*sulI*	+	+	↑	N.A.	Canada	Biswal et al., 2014
	*sulI*	+	+	↓	N.A.	Denmark	Laht et al., 2014
	*sulI*	+	+	↓	+	China	Xu et al., 2015
	*sulI*	+	+	↑	+	China	Yang et al., 2014
	*sulI*	+	–	↓	N.A.	United States	Bergeron et al., 2015
	*sulII*	+	+	↑	N.A.	Canada	Biswal et al., 2014
	*sulII*	+	+	↓	+	China	Yang et al., 2014
	*sulIII*	+	+	↓	+	Canada	Biswal et al., 2014
Multidrug efflux pump genes	*mdtF*	+	+	↓	+	China	Zhang et al., 2011
	*mdtG*	+	+	↓	+	China	Zhang et al., 2011
	*mdtH*	+	+	↓	+	China	Yang et al., 2014
	*mdtN*	+	+	↓	+	China	Yang et al., 2014
	*mexB*	+	+	↓	+	China	Yang et al., 2014
	*mexD*	+	+	↓	+	China	Yang et al., 2014
	*mexF*	+	+	↓	+	China	Yang et al., 2014

*N.A., not analyzed; N.D, no difference.*

Hospital wastewater is a particular risk as it may contain not only pathogenic single- and multi-drug resistant (MDR) bacteria, as detailed in the previous section but also relatively high concentrations of antibiotic compounds. A high percentage of administered antibiotics are not metabolized in humans and are thus excreted into the sewerage (Sabri et al., 2018). Rodriguez-Mozaz et al. (2015) analyzed a broad range of antibiotics including *β-lactams*, *lincosamides*, *macrolides*, *quinolones/fluoroquinolones*, *sulfonamides*, *tetracyclines*, *dihydrofolate reductase inhibitors*, and *nitroimidazoles* and ARGs released from hospitals and urban wastewaters, their removal by a WWTP effluent and their influence on a receiving river. The results show that antibiotics were detected at high concentrations in downstream river samples with antibiotics such as *ofloxacin* reaching concentrations up to 131.0 ng/L while not being detected upstream of the WWTP discharge. Moreover, *ciprofloxacin* and *sulfamethoxazole* had almost 10-fold higher concentrations in downstream than upstream of the WWTP discharge. Studies indicate that the presence of incompletely degraded antibiotic compounds may exert biological selection pressure for the development of ARGs and provide a breeding ground in WWTPs for HGT between bacteria (Zhang et al., 2009; McKinney and Pruden, 2012; Bouki et al., 2013; Sharma et al., 2014) and propagation of resistance genes (Davies and Davies, 2010).

During wastewater treatment, antibiotics, other pharmaceutical residues, and heavy metals present in the wastewater are in continuous contact with bacteria, leading to the potential selection pressure for resistance genes (Zhang et al., 2009; Ding and He, 2010; Bouki et al., 2013). It is difficult to determine a safe concentration of antibiotics in wastewater as results disagree on whether or not antibiotic concentrations lower than the minimum inhibitory concentrations (MIC) cause selection of ARGs. Gullberg et al. (2011) competed for resistant strains against susceptible strains in monoculture with different antibiotic concentrations. The result showed that the resistant strains have a selection advantage even in subminimal inhibitory concentrations and outperform the susceptible strains (Gullberg et al., 2011; Andersson and Hughes, 2014). On the other hand, a recent study by Klümper et al. (2019) suggests that a diverse bacterial community in a mixed culture may select against resistance. Resistant and non-resistant (otherwise isogenic) focal strains (Escherichia *coli*) cultivated together with a pig fecal community, exhibited more than one order of magnitude higher minimal selection concentration for *gentamicin* or *kanamycin*. For the *gentamicin* resistant focal strain, reduced growth was observed due to higher fitness cost for a range of *gentamicin* concentrations (0–10 μg/ml), indicating that resource limitations have a stronger impact on resistant phenotypes (Gómez and Buckling, 2011; Wale et al., 2017; Klümper et al., 2019). However, at very high gentamicin concentrations (100 μg/ml) only resistant strains could grow. The same behavior was observed under intermediate *kanamycin* concentrations (0–20 μg/ml), the susceptible strain did again show improved growth compared to the resistant strain when co-cultured with the pig fecal community. These findings are in accordance with results from the study of Galera-Laporta and Garcia-Ojalvo (2020), where susceptible Bacillus *subtilis* and *E. coli* were cultivated exposed to *ampicillin* separately and in mixed culture. Cultivated separately, *B. subtilis* was able to grow after a lag phase, while *E. coli* died. Cultivated in a mixed culture the two strains displayed reversed reactions to *ampicillin*. The protective effect of the community might play a role and further experimental effort to evaluate the risk of sub-minimal inhibitory concentrations are required.

Heavy metals and some organic compounds, such as quaternary ammonium compounds (QAC), monoaromatic hydrocarbons (MACH), anti-fouling agents and detergents can increase the selective pressure for ARGs through co-selection (Schlüter et al., 2007; Tuckfield and McArthur, 2008; Di Cesare et al., 2016). Two mechanisms for co-selection are normally distinguished: Co-resistance and cross-resistance. Co-resistance refers to the presence of resistance against more than one class of antibiotics in the same bacterial strain. It occurs due to the physical link between different resistance genes, that are placed together, for example on a plasmid, where the selection of resistance to one of the genes leads to resistance to others. Heavy metal resistance genes (HMRGs), especially against zinc and copper, have been shown to increase the rate of AR dissemination by co-resistance (Yazdankhah et al., 2014). Another example is the co-resistance of *qac* genes encoding for efflux pumps against QAC and MACH; the *qac* genes are typically located on MGEs (plasmids and transposons), often together with ARGs (Jiao et al., 2017). In cross-resistance, however, one single resistance mechanism confers resistance to an entire class of compounds, antibiotics and/or other toxicants (Baker-Austin et al., 2006). For example, if two different antimicrobials are present and both have a common strategy to attack the cell, resistance developed against one will be effective against both i.e., the resistance gained for one compound confers resistance for another compound. An example of cross resistance is multi-drug resistance pumps that can export both metals and antibiotics (Baker-Austin et al., 2006). Thus, co-selection is a plausible explanation for the persistence of some ARGs even when antibiotics are not present (Zhang et al., 2018) and both co- and cross-resistance have an important impact on the antibiotic resistance selection in different environments (Stepanauskas et al., 2005; Knapp et al., 2017).

## Antibiotic Resistance Gene Removal in Wastewater Treatment Plants

There are many treatment techniques used in WWTPs that have varying potential to remove organic matter, nitrogen, phosphorous, pollutants and pathogens from wastewater. However, the mechanisms and efficacy of these techniques to remove antibiotics, ARB and ARGs remain mostly unexplored. This section aims to look at the existing situation for the removal of ARB and ARGs from wastewater and sludge in WWTPs.

### Removal From Wastewater

The operation of redox gradient aerobic, anoxic and anaerobic activated sludge reactors and their sequence in a WWTP affects the removal of ARB and ARGs (Christgen et al., 2015; Du et al., 2015; Szekeres et al., 2017). Du et al. (2015) found that anoxic and anaerobic treatment reduced the concentration of ARGs in wastewater, whereas aerobic treatment increased the concentration. The same has been observed by Pei et al. (2007) who proposed that the difference is related to lower growth rates in anaerobic and anoxic compartments compared to aerobic. However, Christgen et al. (2015) have compared three different wastewater treatment strategies; anaerobic, aerobic, and anaerobic–aerobic sequence bioreactors (AAS) in terms of energy use, treatment performance, and ARG abundance. They reported an opposite effect that aerobic bioreactors and AAS bioreactors had higher ARG removal efficiencies than anaerobic bioreactors alone. The AAS bioreactors showed higher removal of ARGs (>85%), compared to separate aerobic (83%) and anaerobic (62%) treatment systems (Christgen et al., 2015). The authors concluded that even though none of these systems were perfect for ARG removal aerobic and AAS were superior to anaerobic bioreactors. Additionally, results suggested that due to lower energy consumption (32% less) AAS systems were seen to be a promising treatment alternative. Moreover, temperature also plays a role in the removal of ARGs showing higher removal at 20°C than at 4°C (Pei et al., 2007), and aerobic treatment may remove more of some types of ARGs than anaerobic at 20°C.

Membrane bioreactors (MBRs) are potentially much better at removing ARB and ARGs than traditional activated sludge reactors. This is because MBRs are better at removing bacteria in general, due to the extra filtration of the effluent through the membrane (Pauwels et al., 2006). The previously mentioned study by Du et al. (2015) reported that the concentration of ARGs throughout a sequence of treatment steps declined proportionally more in the final treatment in an MBR than it did in any of the prior treatment steps in aerobic and anoxic/anaerobic reactors. The MBR showed more than 5 log_10_ units gene copies/100 ml removal of *tetG*, *tetW*, *tetX*, and *sulI* resistance genes, mostly due to filtration (pore size 0.1–0.4 μm) (Du et al., 2015; Hiller et al., 2019). Research by Kappell et al. (2018) has similarly shown the effectiveness of anaerobic MBRs with ARG removals of up to 3.6 log_10_.

Schwermer et al. (2018) investigated the efficiency of two WWTPs in the removal of *ampicillin*, *sulfamethoxazole*, *ciprofloxacin*, and *tetracycline* resistant *E. coli*. The two WWTPs employed a biofilm process and a conventional activated sludge treatment process, respectively. By physical and chemical treatment strategies in WWTP, the percentage of resistant *E. coli* was reduced but full disinfection was not achievable. However, in both conventional activated sludge and the biofilm processes, the percentage of cultivable resistant *E. coli* did not show a considerable decrease in addition to the physical and chemical treatment steps. Moreover, the effluents were also subjected to ultrafiltration (UF) and the total removal effectiveness of *E. coli* in both WWTPs with UF was >4.2 log. Although the ability of DNA to pass through membranes was mentioned by the authors, they stated that membrane filtration processes can provide an additional barrier and post-treatment alternative for the effluent in order to reduce ARB and ARG release by WWTP effluents. Other membrane filtration processes that can be used as post treatment methods include microfiltration (MF) and reverse osmosis (RO). While the effectiveness of MF efficiency against ARB and ARGs has been studied (Riquelme Breazeal et al., 2013), the application of RO, alone or combined with other methods, has yet to be investigated in detail (Schwermer et al., 2018).

Constructed wetlands (CWs) are engineered aquatic systems with very diverse microbial communities and are used to treat wastewater by the same biogeochemical processes dominant in natural wetlands (Doherty et al., 2015; Lv et al., 2017). They are, however, mostly relevant for cases where the total amount of wastewater is relatively low, or for wastewater with lower amounts of organic matter, e.g., urban and agricultural runoff or post treatment of effluents from conventional treatment plants, rather than raw sewage (Zhang et al., 2018; Liu et al., 2019). Their ability to remove ARB and ARGs have brought CWs to attention. CW’s removal mechanisms are dependent on different conditions such as phyta and substrate types together with the physical design of the CW itself (Liu et al., 2019). Li et al. (2019) investigated removal efficiencies for antibiotic and ARG in riverine constructed wetlands. Their results showed that one constructed wetland had 46 and 80% removal efficiency for antibiotics and ARGs, respectively, while another wetland had 70 and 88% removal efficiency, respectively. The difference in efficiencies was associated with antibiotic concentrations in the influent into both wetlands and the scale of the wetland, indicating that the presence of sub-inhibitory levels of antibiotics increases the selective pressure for resistance (Li et al., 2019). In a different study, Chen et al. (2019) designed four different hybrid constructed wetlands. Two horizontal sub-surface flow (HSSF) CWs, one with and one without artificial aeration, and two vertical sub-surface flow (VSSF) CWs, again one with and one without artificial aeration. These four CWs were tested for their ability to remove antibiotics and ARGs. Efficiencies between 87 and 95% for total antibiotic removal, and between 88 and 99% for total ARG removal, were reported. The authors found that the hybrid constructed wetlands with artificial aeration compared to CWs without artificial aeration had higher removal efficiencies of ARB and ARGs, together with higher removal rates of organic carbon, ammonia, nitrogen, and phosphorous.

Several recent studies have investigated the removal of ARGs in WWTPs by chemical disinfection processes, such as chlorination and advanced oxidation processes (AOPs) including ozonation and UV. It showed that these processes can significantly decrease the occurrence of ARGs and pathogenic microorganisms in WWTP effluents (Luczkiewicz et al., 2011; Zhuang et al., 2015; Hiller et al., 2019). Zhuang et al. (2015) reported chlorine disinfection resulted in 1.654–2.28 log_10_ reduction, and UV irradiation resulted in 0.80–1.21 log_10_ reduction of ARGs under economically suitable operational conditions. Although ozonation disinfection achieved 1.68–2.55 log_10_ reduction of ARGs, the authors in the same study advised against the use of this process due to excessive operational costs. Contrary to this, Alexander et al. (2016) indicated that even though ozone treatment can reduce the *erythromycin* resistance gene (*ermB*) by 2 orders of magnitude, ARGs against *vancomycin (vanA)* and *imipemem (blaVIM)* increased within the surviving wastewater bacterial population. Luczkiewicz et al. (2011) showed that ultrafiltration, ozonation, and UV irradiation can reduce the amount of fecal coliform bacteria in wastewater by more than 99%, but there was a slightly higher percentage of ARGs containing bacteria among the bacteria surviving disinfection. They found that of coliforms grown from water samples taken before and after disinfection, 47–60% of *E. coli* isolates were resistant after disinfection compared to 42–50% of isolates before and that 68–90% of *Enterrococus* spp. isolates had resistance after treatment compared to 68–85% before. Recently, using a the combination of two or more AOPs (like Fenton’s oxidation reaction, UV/H_2_O_2_, solar/H_2_O_2_, photo-Fenton process, TiO_2_ photocatalyst and ionizing radiation) have been shown to be effective in the removal of refractory organic compounds (like antibiotics) in secondary effluents (Rizzo et al., 2013; Zhang et al., 2016b). Karaolia et al. (2014) investigated a solar-driven Fenton oxidation that may eliminate ARB. Mccullagh et al. (2007) reported that the utilization of a UV-TiO_2_ photocatalyst AOP inactivated a diverse array of bacterial, viral, and protozoal organisms from water and wastewater. While AOPs represent a potential way to remove antibiotics and thus prevent antibiotic resistance, they are not widely used due to their operational costs (Qiao et al., 2018). Table 2 shows different treatment techniques and their effectiveness in removing different ARGs and pathogens from wastewater under different conditions. Additionally, Table 3 summarizes the antibiotic elimination efficiencies in different wastewater treatment units. Both tables include information on different treatment techniques categorized in physical, biological, and chemical processes.

**TABLE 2:** Antibiotic resistance gene (ARG) and pathogen elimination efficiencies of different treatment technologies.

Treatment technologies	Target ARGs	ARG elimination efficiency	Pathogen elimination efficiency	References
**Physical processes**				
Membrane separation	*floR*, *sulI*, and *sulII*	∼98%	99.9%	Ren et al., 2018
Soil aquifer treatment	*bla*_*TEM*_ and *qnrS*	>2 logs	1.2–6.9 logs	Sharma and Kennedy, 2017; Elkayam et al., 2018
**Biological processes**				
Anaerobic–aerobic seq. bioreactor (AAS)	*Sulfonamide*, *chloramphenicol*, *aminoglycoside*, *tetracycline*, *β-lactam resistance genes*	>85%		Christgen et al., 2015; Thwaites et al., 2018
Aerobic bioreactor		83%		Christgen et al., 2015
Anaerobic bioreactors		62%	18%	Christgen et al., 2015; Zhang et al., 2017
Membrane bioreactor	*bl*and*M-1*, *blaCTX-M-15*, *and blaOXA-48*	2.76–3.84 logs	2.7–5.6 logs	Cheng and Hong, 2017; Harb and Hong, 2017
	*sulI*, *sulII*, *tetC*, *tetX*, *ereA*, and *int1*	0.5–5.6 logs	–	Zhu et al., 2018
	*sul1*, *tetG*, *tetW*, and *tetX*	5 log/100 ml	–	Du et al., 2015
	*ermB*, *tetO*, *sulI*, and *intl1*	≤3.6 log	–	Kappell et al., 2018
CW-surface flow	*sulI*, *sulII*, *sulIII*, *tetA*, *tetB*, *tetC*, *tetE*, *tetH*, *tetM*, *tetO*, *tetW*, *qnrB*, *qnrS*, and *qepA*	77.8% in summer, 59.5% in winter	0.96–4.46 logs	Fang et al., 2017; Shingare et al., 2019
CW-horizontal subsurface flow	*intl1*, *sulI*, *sulII*, *dfrA*, *aac6*, *tetO*, *qnrA*, *blaNMD1*, *blaKPC*, *blaCTX*, and *ermB*	-145.6 to 98.9%	0.7–5.51 logs	Yi et al., 2017; Chen et al., 2019; Shingare et al., 2019
CW-vertical subsurface flow	*tet genes* and *intI1*	33.2–99.1%	0.5–2.84 logs	Huang et al., 2017b; Shingare et al., 2019
CW	*sulI*, *sulII*, *tetA*, *tetC*, *dfrA1*, *dfrA12*, *dfrA13*, *ermB*, and *bla_*PSE*__–__1_*	80.2 and 87.5%	–	Li et al., 2019
Hybrid CWs	*sulI*, *sulII*, *tetG*, *tetO*, *ermB*, *qnrS*, *qnrD*, *cmlA*, and *floR*	87.8–99.1%	0.71–4.8 logs	Chen et al., 2019; Shingare et al., 2019
**Chemical processes**				
Chlorination	*sulI*, *tetG*, and *intI1*	1.65–2.28 logs	∼3 logs	Zhuang et al., 2015; Furst et al., 2018
	*sulI*, *tetX*, *tetG*, and *intI1*	1.20–1.49 logs	–	Zhang et al., 2015
	*tetA*, *tetB*, *tetC*, *sulI*, *sulII*, *sulIII*, *ampC*, *aph*(*2’*)-*Id*, *katG*, and *vanA*	Enhancement	–	Liu et al., 2018
UV	*sulI*, *tetG*, and *intI1*	0.80–1.21 logs	30 min, 254 nm, 2.0 ± 0.3 logs	Zhuang et al., 2015; Sousa et al., 2017
	*tetW*	0.00–1.89 logs	–	Sullivan et al., 2017
	*tetX*, *sulI*, *tetG*, and *intI1*	0.36–0.58 logs	–	Zhang et al., 2015
Ozonation	*tet* genes and *sul* genes	<49.2 and <34.5%	30 min, 2.1 ± 0.5 logs	Sousa et al., 2017; Zheng et al., 2017
Photocatalytic oxidation	*sul1*, *tetX*, and *tetG*	2.63–3.48 logs (pH = 3.0) 1.55–2.32 logs (pH = 7.0)	2–3 logs	Zhang et al., 2016b; Moreira et al., 2018
Fenton’s oxidation reaction	*sul1*, *tetX*, and *tetG*	2.58–3.79 logs (pH = 3.0) 2.26–3.35 logs (pH = 7.0)	<LOQ*	Zhang et al., 2016b; Moreira et al., 2018

*LOQ, Limit of Quantification.

**TABLE 3:** Antibiotic elimination efficiencies in different treatment units.

Treatment technologies	Antibiotics	Antibiotic elimination efficiency	References
**Physical processes**			
Membrane separation	SA, ML	5–28%	Sahar et al., 2011
Soil aquifer treatment	SA	68.2–88.9%	Qin et al., 2020
	ML	90.1%	Fang et al., 2017
**Biological processes**			
Anaerobic–aerobic seq. bioreactor (AAS)	–	–	–
Aerobic bioreactor	SA	>95%	Qian et al., 2020
Anaerobic bioreactors	SA	∼2%	Qian et al., 2020
	TC, SA	>90%, 30–98%	Cheng et al., 2020
Membrane bioreactor	SA	87.4%	Song et al., 2017
	FQ	81.1%	
	TC	83.8%	
	ML	14.3%	
CW-surface flow	BL, SA, FQ, TC, ML	–67 to 100%	Liu et al., 2019
CW-horizontal subsurface flow	BL, SA, FQ, TC, ML	–46 to 100%	Liu et al., 2019
CW-vertical subsurface flow	BL, SA, FQ, TC, ML	20–100%	Liu et al., 2019
Hybrid CWs	SA, FQ, TC, ML	43 ± 32%	Ávila et al., 2014
**Chemical processes**			
Chlorination	BL	97–100%	Li and Zhang, 2011
	SA	73–100%	
	FQ	50–74%	
	TC	39–83%	
	ML	43–53%	
UV-254 nm	SA	51%	De la Cruz et al., 2012
	FQ	48–65%	
	ML	0%	
	SA, FQ	>99%, 90 min	Michael et al., 2020
Ozonation	BL, SA, FQ, ML	100%, O3 = 14–42 mg	Paucar et al., 2019
	TC	86.4–93.6%, O3 = flow rate 0.5 L/min	Wang et al., 2018
Photocatalytic oxidation	SA	∼46%, 300 min, Q_*UV*_ = 42 kj/L	Michael et al., 2020
	FQ	>99%, 60 min, Q_*UV*_ = 8 kj/L	
	SA, FQ, TC	100%, 90–100%, 100%	Palominos et al., 2008; Kansal et al., 2014; Espíndola et al., 2019; Sandikly et al., 2019
Fenton’s oxidation reaction	BL	100%, H_2_O_2_/Fe^2+^ = 2–150 μM, pH = 2–4	Elmolla and Chaudhuri, 2009
	SA	74%, H_2_O_2_/Fe^2+^ = 2.9 μM, pH = 3–6	Qian et al., 2020
	FQ, ML	With citric acid 95%, H_2_O_2_/Fe^2+^ = 1.75 μM, pH = 3	Macku’ak et al., 2015

*BL, β-lactam; SA, sulphonamide; FQ, fluoroquinolones; TC, tetracyclines; ML, macrolides.*

All the studies conducted in the literature together with the information presented in Tables 2, 3, suggest that the wastewater to be treated should be analyzed for antibiotics and ARGs, in addition to the standard wastewater characterization parameters. The WWTP should be designed using this characterization specially tailored for the needs of the specific wastewater ensuring the removal of antibiotics and ARGs to avoid the spread of antibiotic resistance in the receiving water body. However, even though the removal efficiency of disinfection processes is very high it is not possible to avoid secondary treatment to cut cost, since the organic matter in the wastewater act as precursors of disinfection by-products. Additionally, the secondary treatment also decreases the suspended solids concentration, which is a key parameter for UV disinfection. As a further treatment step, membrane filtration systems might be used to remove the remaining ARG and ARB, and CW can be considered as a post treatment step for effluents in smaller settings. Finally, MBRs that combine biological treatment and membrane filtration make good alternatives for ARB and ARG removal.

### Removal From Sludge and Biosolids

Biological wastewater treatment relies on the growth of bacteria and other microorganisms and subsequent flocculation and settling of aggregated biomass. At a steady state, excessive biomass is removed (so called sludge wasting usually done through the underflow from secondary clarifiers) together with other solids that are collected in skimmers and primary clarifiers. Several unit operations reduce water content, stabilize, and treat the discarded sludge before it is disposed or recycled. Biosolids from WWTPs are typically applied to agricultural land as fertilizer, disposed of to landfills, or incinerated (United States Environmental Protection Agency, 2003; Tchobanoglous et al., 2014; Collivignarelli et al., 2019).

Unsurprisingly, most of the resistant bacteria and resistance genes that arrives with the sewage and that grows and propagates through a treatment plant end up in the settled sludge (Munir et al., 2011; Calero-Cáceres et al., 2014; Yuan et al., 2019). Studies examining municipal WWTPs without advanced sludge treatment in the United States (Munir et al., 2011; Gao et al., 2012) and in China (Chen and Zhang, 2013; Wen et al., 2016; Yuan et al., 2019) have shown that although the plants are able to reduce the abundance of resistance genes and resistant bacteria in their effluents by 2–4 orders of magnitude, the amount of resistance genes and resistant bacteria in the biosolids from these plants are of the same order of magnitude as in the inflow sewage (around 10^8^–10^10^ copies of *tetW* and *tetO* resistance genes per 100 ml sample and 10^6^–10^8^ CFU of *tetracycline* resistant bacteria per 100 ml sample).

Supplementary Table 1 gives an overview of reported levels of bacteria, resistant bacteria, and resistance genes in biosolids after sludge treatment at WWTPs around the world. The table also includes extended information about plant type, treatment process, sludge sources, and final application of the biosolids. Conventional sludge treatment methods that simply thickens and dewaters sludge by gravity thickening, belt pressing, centrifugation, or other mechanical methods are not effective at removing resistance genes (Supplementary Table 1). Further anaerobic or aerobic digestion of the sludge is also in many cases not enough to substantially reduce the number of resistant bacteria and genes.

Heat drying, which involves reducing the moisture content to below 10% by direct or indirect contact with hot gases (Tchobanoglous et al., 2014), and advanced lime stabilization, which involves the addition of alkali (lime) to increase pH in combination with other treatments like pasteurization or heat drying, are more effective at removing resistance. Heat drying and advanced lime stabilization reduce the density of bacteria in biosolids, and thus also the density of resistant bacteria, to levels similar to and in most cases below what is typical for soil (Supplementary Table 1). This is due to the high temperatures and/or pH that are reached in the processes. The density of resistance genes is on the other hand in many cases still higher than what is typical for unfertilized soil. However, it has been suggested and observed that the genes have lower stability after treatment as many are trapped within dead microorganisms (Lau et al., 2017; Murray et al., 2019). In a study that measured the density of resistance genes in the soil directly after application of biosolids, the authors found that the abundance of resistance genes 2 h after application was remarkably low in soil amended with heat-dried biosolids (Lau et al., 2017). Similar results have also been found for soil amended with biosolids pasteurized at more than 70°C for a period of 30 min in a lab scale experiment (Burch et al., 2017). It seems that many of the resistance genes are rapidly destroyed when they come in contact with soil and moisture.

The N-rich biosolids produced through the N-Viro treatment used at Thorold, Ontario (Supplementary Table 1) have particularly low levels of resistance genes. Murray et al. (2019) found that out of 41 selected genes associated with resistance and HGT, 38 were below the detection limit and the remaining 3 were below the quantification limit. The reason is that the pH is so high that double stranded DNA denatures (Murray et al., 2019).

Pyrolysis is another treatment method that consistently reduces the density of resistance genes in biosolids to below what is found in pristine soil in nature. Pyrolysis is not a common biosolid treatment technique today and has many of the same disadvantages as incineration. It has high capital and operating cost, and requires highly skilled operating and maintenance staff, compared to the simpler dewatering, stabilization, and heat drying methods (Tchobanoglous et al., 2014; Carey et al., 2016). It is also energy intensive, but it can potentially be used as a refinement step in treatment plants that already use heat drying, as the added energy cost of pyrolysis is reported to be low compared to the energy already invested in drying the biosolids (McNamara et al., 2016). The benefit of pyrolysis over incineration is that more organic content and nutrients remain in biochar than in incinerated ash, giving biochar a higher fertilizer potential—biochar has an NKP content of 6-13-0 vs. 0-6-2 for incinerated ash (Carey et al., 2016). They both, however, have the risk of containing high levels of heavy metals, which are concentrated in the product during the production process (Carey et al., 2016).

From the results combined in Supplementary Table 1, it is worth noting that the density of remaining resistance genes after a specific biosolids treatment method can vary with more than an order of magnitude between facilities (Supplementary Table 1). This may be due to differences in the sludge loading or their operation, but also because the methods and protocols for quantification have different sensitivities and efficiencies for extracting and measuring the absolute concentration of genes (Feinstein et al., 2009; Taylor et al., 2019; Yuan et al., 2019).

The trend seen from the numbers in Supplementary Table 1 is that further treatment beyond digestion is needed to reduce the density of resistant bacteria to levels comparable to or below what is found in soils. The trend coincides comparatively well with the grouping of sludge treatment methods used in the biosolids regulation of the United States (40 CFR Part 503) (United States Environmental Protection Agency, 1994, 2003). Treatment processes are categorized into “processes to significantly reduce pathogens” (PSRP) and “processes to further reduce pathogens” (PFRP). PSRP includes the first set of treatment methods after thickening/dewatering, i.e., aerobic digestion, anaerobic digestion, air drying, composting, and limes stabilization, with specific requirements to process parameters such as time, temperature, and pH (United States Environmental Protection Agency, 1994, 2003). PFRP includes further treatments that use heat or radiation to purposefully kill pathogens, i.e., heat drying, heat treatment, pasteurization, beta- or gamma-ray irradiation, and also composting and thermophilic digestion if the temperature is kept over 55°C for a specified number of days (United States Environmental Protection Agency, 1994, 2003). The PSRP and PFRP grouping are in the United States are used together with bacteria density limits (fecal coliforms or Salmonella) to regulate land application of biosolids. However, the current regulations only require PSRP treatment or an average fecal coliform density below 2⋅10^6^ CFU/g for agricultural use (class B biosolids), and there is no specific mention of either resistant bacteria or resistance genes (United States Environmental Protection Agency, 1994, 2003). Similarly, there are currently no specific limits on resistant bacteria or resistance genes for biosolids in the European Union (Eur-Lex, 2018; Collivignarelli et al., 2019). The European Union directive 86/278/EEC (2018), which regulates the application of biosolids in the EU, does not specify any limits on pathogen content, but several member states have national regulations with limit values for indicator bacteria [typically Salmonella and some type(s) of fecal bacteria] (Collivignarelli et al., 2019).

More and more studies are linking the application of biosolids to higher levels of resistant bacteria and genes in agricultural soil (Ross and Topp, 2015; Gondim-Porto et al., 2016; Burch et al., 2017; Lau et al., 2017; Murray et al., 2019). However, the resistance levels decrease with time after application (Marti et al., 2014; Rahube et al., 2014; Ross and Topp, 2015; Burch et al., 2017; Lau et al., 2017; Murray et al., 2019), and the current evidence for gene transfer to crops and animals remains inconclusive (Marti et al., 2013b; Rahube et al., 2014; Lau et al., 2017; Murray et al., 2019; You et al., 2020). Current regulations in the US and the EU do include time restrictions from application to harvesting and/or grazing (United States Environmental Protection Agency, 1994, 2003; Eur-Lex, 2018; Collivignarelli et al., 2019). Implementation of limits for the density of resistant bacteria and resistance genes to the regulations for biosolids should also be considered. Limits on the density of resistance genes can be difficult to implement, as measurement methods for gene amounts have varying sensitivity and accuracy (Feinstein et al., 2009; Taylor et al., 2019; Yuan et al., 2019). The density of resistance genes can furthermore be an inconsistent factor for risk alone because of the difference in stability and transfer potential between genes in living bacteria, genes in dead bacteria, and free and adsorbed genes outside of bacteria. Significant risk reduction can be achieved merely by stricter limits on the general density of bacteria, e.g., as for biosolids of class A today (Murray et al., 2019). Treatment operations that consistently reach these limits are already implemented technologies at many WWTPs. Stricter limits must, however, be weighed against the implications they will have for the overall use of biosolids as fertilizer and soil improvement. Moreover, limits and regulations for biosolids must be harmonized with other biological fertilizers such as manure, which is also known to contain high levels of resistance (Marti et al., 2013b, 2014; Ross and Topp, 2015; Nõlvak et al., 2016). Stricter regulations can lead to more incineration and less reuse, an effect that cannot be disregarded in the context of a sustainable and circular economy.

## Modeling Antibiotic Resistance in Wastewater Treatment Plants

Mathematical models formulated from a mechanistic or holistic understanding of microbial and biogeochemical interactions in aquatic systems have advanced our understanding of dynamics in technical and natural systems (Simon et al., 2002; Benedetti et al., 2013). Mathematical models describing the processes involved in the treatment and biodegradation of wastewater have already successfully been developed and established as standard, well used tools within the WWTP community (Gernaey et al., 2004; Solon et al., 2019). Such models are, as mathematical models in general, functional tools for *a priori* model and hypothesis testing, and *a posteriori* data analysis and performance evaluation. The standard WWTP models are the so-called activated sludge models (ASM1, ASM2, ASM2d, ASM3, and variants), which have been applied for research and process performance evaluations, as well as for the design of new WWTPs (Henze et al., 2000; Van Loosdrecht et al., 2015). These models include the major WWTP processes of biomass growth, carbon oxidation, nitrification, denitrification, and phosphorus removal. None of the standard models, however, include the occurrence or spread of antibiotic resistance among bacteria in WWTPs.

Several mathematical models for the spread of antibiotic resistance in bacteria populations have been proposed, although mainly in theoretical, or simplified, environmental settings (Birkegård et al., 2018). This includes models of the spread of resistance in axenic cultures of bacteria (Tremblay and Rose, 1985; Imran and Smith, 2007; Svara and Rankin, 2011), and a few models that include spread through more than a single strain (Clewlow et al., 1990). There are also models that deal with the dynamics of antibiotic resistance in relation to antibiotic concentrations and distinguish the type of resistance mechanism (Bootsma et al., 2012; Krzyzanski and Rao, 2017); and finally, models that deal with the spread between hosts of bacteria, i.e., in an epidemiological setting (Spicknall et al., 2013; Levin et al., 2014).

The development of mathematical models that combine the biodegradation processes and population dynamics of microorganisms in a WWTP with the presence of antibiotic compounds, ARGs, and the spread of antibiotic resistance in the populations through HGT is still in its early stages. Attempts to combine the use of WWTP and ARG models are few and limited to early-stage developments. There have, however, been attempts to combine antibiotic degradation dynamics with the traditional activated sludge models (ASM-X by Polesel et al., 2016) to assess degradation kinetics of antibiotics and other pharmaceuticals in WWTPs. Few examples exist of models that have been set up to address the effect of antibiotic resistance in realistic environments that are partly similar to WWTPs (Hellweger et al., 2011; Hellweger, 2013; Baker et al., 2016). Baker et al. (2016) modeled the spread of antimicrobial resistance in a slurry tank that collects and stores fecal and urinary waste from cows at a dairy farm. Their model includes most processes that should be considered to capture both population dynamic and resistance spread, i.e., cellular growth and death processes, HGT, segregation loss, antibiotic concentration (to capture selection pressure), slurry inflow and fitness cost. We think that this model structure with the addition of the treatment processes from the ASM models can serve as a basis for a model suitable for a WWTP environment. Baker et al. (2016) parameterized their model based on their experimental data and data from the literature and showed through sensitivity analysis that gene transfer rate is one of the most important parameters for the spread of resistance. Hellweger et al. (2011) and Hellweger (2013) used a mathematical model to test if observed concentrations of antibiotics and densities of *tetracycline* resistant bacteria in the Poudre River in Colorado could be explained by different scenarios for how resistant exogenous bacteria that arrive at the river grow and exchanges genes with indigenous bacteria. They showed that the observed data could not be explained by a scenario with high input of exogenous resistant bacteria to the river without growth in the river itself; their model suggested that there has to be the growth of resistant bacteria and thus maintenance of the resistance gene in the river itself, is most likely due to soft selection pressure from low concentrations of tetracycline (Hellweger et al., 2011).

Wastewater treatment plants are highly complex systems with mixed cultures of microorganisms, and a wide range of modeling approaches, including individual-based models (IbMs), are needed to understand the functioning of such plants from micro to macro scale. Deterministic population-level models, like the classical ASM models, allow for studying the average behavior of systems, e.g., the overall dynamics of populations and concentrations in the plant reactors. However, they may miss some important individual effects on biological processes rates in the bacterial community. Population-level models do not account for individual heterogeneity, local interactions, or adaptive behavior. IbMs do on the other hand treat bacteria as single cells, as discrete entities, and might be better suited to account for the spread of resistance and can potentially overcome these limitations that arise from population model design. HGT is a micro-level process. For example, conjugation of resistance plasmids, as this is a discrete event between individual cells, happens when an individual donor bacterium and a recipient bacterium are close enough in space that a pilus from the donor can attach to the recipient and bring them together (Seoane et al., 2011). IbM of conjugation mechanism allows presenting the intrapopulation variability, to capture the changes that occur during the coupling process (Merkey et al., 2011; Seoane et al., 2011). Moreover, local variations in population density, e.g., flocculation, plays a role in the spread of resistance (Merkey et al., 2011), and the description of actions on the level of the single organism in the model may thus be needed to explain the total population development (Breckling, 2002; Hellweger et al., 2016). IbMs can determine the relevance of a specific interaction or location for the overall behavior of the biofilm Therefore, IbMs can give insights into the emergence of antibiotic resistance from biofilms to aquatic environments. Moreover, the combination of different level models, population and individual, can provide quantitative analysis of the spread of antibiotic-resistant bacteria.

Although mathematical models can be powerful tools and the ASM models have been very successful for understanding and developing treatment processes, mathematical modeling approaches have so far not yet been able to help improve our understanding of the conditions that drive maintenance, spread or extinction of ARGs or ARB in WWTPs. The main weakness of many of the proposed models of antimicrobial resistance is the lack of experimental data available to parameterize and validate the models (Birkegård et al., 2018). Any extensions of the standard WWTP models to include resistant and non-resistant bacteria, the presence of ARGs, different mechanisms for HGT, and concentrations of antibiotics, should include considerations on how to experimentally measure associated process rates and concentrations. An integrated AR-WWTP model designed -with this in mind- can become a promising tool for theoretical and diagnostic studies of ARG spreading, and it can be of help in identifying which mechanisms and factors that are the most important for the spread of resistance under different circumstances. That is, in evaluating which operational conditions or parameter values that can minimize spread, and which parameters are key drivers.

## Future Directions and Conclusion

In this review, we assessed the causes and the mechanisms for the spread of ARGs, together with their occurrence, transfer, and potential removal in WWTPs. While the issue of antibiotic resistance could never have completely been prevented, the current universal problem of resistant bacteria is solely due to anthropogenic activities. Moreover, the absence of regulations and strict monitoring regimes have contributed to the escalation of the occurrence of antibiotic resistance in the environment. The research shows that neither conventional nor advanced WWTPs are efficient enough to completely remove ARGs and ARB from water environments, but that more advanced treatment methods perform better. Advanced post treatment methods like UV, ozonation and oxidation of water effluents, and heat drying, lime stabilization and pyrolysis of biosolids, remove considerably more ARGs and ARB than activated sludge treatment alone but are not without disadvantages like more difficult and complex operation and higher cost. Finally, the following key points are proposed to improve current WWTPs and provide guidance for future application:

(i) In order to reduce the threat of antibiotic resistance, it is advisable to set strict threshold limits for antibiotic release from point sources like hospitals and animal husbandries, together with the thresholds for release of metal residues, biocides, and other pharmaceuticals that drive co-selection of resistance.
(ii) Plans for implementation of more advanced treatment processes should consider the economy and ecology of the whole waterway. It may be more cost effective to employ smaller scale treatment plants with disinfection units at point sources than to redesign and rebuild larger municipal WWTPs.
(iii) Efforts should be made to devise and agree upon standard methods to measure and report ARB and ARG levels to make it easier to compare resistance levels between different countries and at different treatment plants. This will also make it easier to evaluate removal efficiencies of treatment methods and to evaluate the performance of already established treatment plants, which can facilitate the decision process of operators and regulatory agencies of whether additional post-treatment steps are necessary.
(iv) Experimental studies should be combined with mathematical modeling to further examine the mechanisms for the spread and population growth of resistant and non-resistant bacteria in wastewater treatment environments. The effect of different treatment methods and plant operation strategies on the spread of resistance genes should be further studied, including the effect of operating conditions (pH, temperature, COD, BOD) on HGT.

These approaches can provide a further understanding of the processes and mechanisms of spread and can therefore help in the design of WWTPs that are less likely to become breeding grounds for antibiotic resistance, and which function better as final barriers.

## Author Contributions

CU, KMK, and IP-O researched, wrote, and edited the manuscript. KT researched and wrote section “Removal From Sludge and Biosolids” and edited the manuscript. DB researched and designed Table 3. SS, MJ, and GK conceived the review outline. RK provided significant input on wastewater treatment and operation of WWTPs and edited the manuscript. All authors reviewed the manuscript.

## Conflict of Interest

The authors declare that the research was conducted in the absence of any commercial or financial relationships that could be construed as a potential conflict of interest.

## Publisher’s Note

All claims expressed in this article are solely those of the authors and do not necessarily represent those of their affiliated organizations, or those of the publisher, the editors and the reviewers. Any product that may be evaluated in this article, or claim that may be made by its manufacturer, is not guaranteed or endorsed by the publisher.
