# Supplementary material for: A Review on Occurrence and Spread of Antibiotic Resistance in Wastewaters and in Wastewater Treatment Plants: Mechanisms and Perspectives
Source: Front Microbiol. 2021 Oct 11;12:717809. doi: 10.3389/fmicb.2021.717809 (PMC8542863; doi:10.3389/fmicb.2021.717809)
Supplement: Supplementary file 1 [file Table_1.pdf]

Supplementary File: A review on occurrence and spread of antibiotic resistance in wastewaters and in wastewater treatment plants: Mechanisms and perspectives

Cansu Uluseker[1†], Krista Michelle Kaster[1†], Kristian Thorsen[2], Daniel Basiry[1], Sutha Shobana[3], Monika Jain[4], Gopalakrishnan Kumar[1], Roald Kommedal[1], Ilke Pala-Ozkok[1*]

[1] Department of Chemistry, Bioscience and Environmental Engineering, Faculty of Science and Technology, University of Stavanger, Box 8600 Forus, 4036 Stavanger, Norway

[2] Department of Electrical Engineering and Computer Science, Faculty of Science and Technology, University of Stavanger, Box 8600 Forus, 4036 Stavanger, Norway

[3] Department of Chemistry and Research Centre, Aditanar College of Arts and Science, Virapandianpatnam, Tiruchendur, Tamil Nadu, India

[4] Department of Natural Resource Management, College of Forestry, Banda University of Agricultural and Technology, Banda - 210001, India

[†] **These authors have contributed equally to this work**

[*] **Correspondence:**
Corresponding Author
ilke.palaozkok@uis.no

Keywords: antibiotics, antibiotic resistance genes, antibiotic resistant bacteria, spread mechanisms, wastewater treatment plant

Supplementary Table 1: Bacteria, resistant bacteria, and resistance genes remaining in biosolids after different treatment methods

**Sludge/biosolids treatment,**<br><br>**Wastewater plant**	**Resistant bacteria**	**Resistance genes**<br><br>**(total cellular + free DNA)**	**Wastewater treatment process, source of biosolids, sludge treatment, measurement methods and other notes**	**Ref.**
**Belt press dewatering**				
East Lansing, Michigan, USA<br>(cake 18.05% solids)<br><br>*	Total CFU/g $10^{10.0} – 10^{11.7}$<br><br>*tet* resistant CFU/g $10^{7.4} – 10^{8.3}$<br>(16 ug/mL *tet*)<br><br>*sul* resistant CFU/g $10^{8.7} – 10^{9.1}$<br>(50.4 ug/ml *sul*)	Total copies/g $10^{11.5} – 10^{12.1}$<br>(16S rRNA)<br><br>*tetW* copies/g $10^{8.0} – 10^{8.5}$<br>*tetO* copies/g $10^{8.8} – 10^{9.3}$<br><br>*sulI* copies/g $10^{6.9} – 10^{9.2}$	Activated sludge treatment, biosolids from a mix of primary sludge and activated sludge dewatered by belt press only. Bacteria numbers by heterotrophic plate count on R2A media with antifungal cyclohexamide (200 ug/ml), gene copy numbers from qPCR, numbers per gram of biosolids (wet or dry weight not specified), absolute ranges from n = 3 samples taken at different times of the year.<br><br>Biosolids disposed to landfill.	#:<br>(Munir et al., 2011)<br><br>PT&TP:<br>(City of East Lansing Webpages, 2009; Munir et al., 2011)
**Gravity thickening**				
Imlay, Michigan, USA<br>(slurry 1.49% solids)<br><br>*	Total CFU/g $10^{9.5} – 10^{10.0}$<br><br>*tet* resistant CFU/g $10^{7.0} – 10^{9.0}$<br>(16 ug/mL *tet*)<br><br>*sul* resistant CFU/g $10^{8.0} – 10^{9.3}$<br>(50.4 ug/ml *sul*)	Total copies/g $10^{12.6} – 10^{12.7}$<br>(16S rRNA)<br><br>*tetW* copies/g $10^{8.7} – 10^{8.9}$<br>*tetO* copies/g $10^{8.7} – 10^{8.9}$<br><br>*sulI* copies/g $10^{7.5} – 10^{9.4}$	Oxidation ditch treatment, biosolids from unknown sludge mix, gravity thickening only. Bacteria numbers by heterotrophic plate count on R2A media with antifungal cyclohexamide (200 ug/ml), gene copy numbers from qPCR, numbers per gram of biosolids (wet or dry weight not specified), absolute ranges from n = 2 samples taken at different times of the year.<br><br>Biosolids used as fertilizer on agricultural land.	#:<br>(Munir et al., 2011)<br>PT&TP:<br>(Imlay City Webpages, 2020)
**Anaerobic digestion**				
Romeo, Michigan, USA<br>(slurry 7.98% solids)<br>Traverse city, Michigan, USA<br>(slurry 4.85% solids)	Total CFU/g $10^{7.3} – 10^{9.5}$<br><br>*tet* resistant CFU/g $10^{4.6} – 10^{5.7}$<br>(16 ug/mL *tet*)	Total copies/g $10^{12.0} – 10^{12.6}$<br>(16S rRNA)<br><br>*tetW* copies/g $10^{7.8} – 10^{9.6}$<br>*tetO* copies/g $10^{7.1} – 10^{8.4}$	Combined data from the Romeo and the Traverse city plants. Rotating biological contactors (Romeo) with biosolids from unknown sludge mix. Aeration basin and membrane biological reactor (Traverse city) with biosolids from a mix of primary sludge and activated sludge dewatered by a gravity	#:<br>(Munir et al., 2011)<br><br>PT&TP:

*	*sul* resistant<br>(50.4 ug/ml Sul)	CFU/g	$10^{6.7} – 10^{7.2}$	*sulI*	copies/g	$10^{7.6} – 10^{8.2}$	belt concentrator before digestion. Bacteria numbers by heterotrophic plate count on R2A media with antifungal cyclohexamide (200 ug/ml), gene copy numbers from qPCR, numbers per gram of biosolids (wet or dry weight not specified), interquartile ranges from n = 7 samples in total taken from different times of the year (unspecified how many samples from each site). Temperature during anaerobic digestion unspecified.<br><br>Biosolids used as fertilizer on agricultural land.	(Munir et al., 2011; Traverse City/Jacobs, 2018)
Guadarrama Medio, Brunete, Madrid, Spain (cake 16.5% solids)	Total<br><br>*amp* resistant<br>(32 ug/mL Amp)<br><br>Fecal coliforms	CFU/g<br><br>CFU/g<br><br><br>CFU/g	$10^{11.2}$<br><br>$10^{8.8}$<br><br><br>$10^{8.6}$				Activated sludge treatment, biosolids from a mix of primary sludge and activated sludge. Gravity thickening of the sludge before digestion. Two stage digestion with heating. Mechanical dewatering of biosolids by centrifuges and thermal drying. Bacteria numbers by heterotrophic plate count on R2A media with antifungal cyclohexamide (200 ug/ml), fecal coliforms numbers by plate count on TTC medium. Numbers per gram dry weight of biosolids, average numbers from unknown number of samples. Temperature during anaerobic digestion unspecified.<br><br>Biosolids used as fertilizer on agricultural land.	#:<br>(Gondim-Porto et al., 2016)<br><br>PT&TP:<br>(Gondim-Porto et al., 2016; Canal de Isabel II S.A., 2017)
Galt, Ontario, Canada (slurry 4.9% solids)	Total coliforms<br>Fecal coliforms<br>*E. coli*	CFU/g<br>CFU/g<br>CFU/g	$10^{6.5}$<br>§ $< 10^{3.6}$<br>§§ $< 10^{2.3}$	Total (rrnS)<br><br>*sulI*<br><br>*strA*<br>*strB*	copies/g<br><br>copies/g<br><br>copies/g<br>copies/g	$10^{10.1}$<br><br>$10^{9.9}$<br><br>$10^{8.5}$<br>$10^{7.4}$	Activated sludge treatment, biosolids from a mix of primary sludge and mechanically thickened activated sludge. Total coliforms, fecal coliforms, and *E. coli* numbers by plate count on mEndo-LES medium, mFC medium, and MFC-BCIG medium respectively. Gene copy numbers from qPCR. Numbers per gram dry weight of biosolids. Average numbers from n = 3 samples for bacteria count and n = 4 samples for gene numbers. Temperature during anaerobic digestion unspecified.<br><br>Biosolids used as fertilizer on agricultural land and for remediation of mine tailing ponds from mining industry. Landfill as contingency option.	#:<br>(Lau et al., 2017)<br><br>PT&TP:<br>(Lau et al., 2017; Region of Waterloo, 2018)
Galt, Ontario, Canada (cake 30.1% solids)				Total (rrnS)	copies/g	$10^{8.4}$	Activated sludge treatment, biosolids from a mix of primary sludge and mechanically thickened activated sludge. Mechanical dewatering of biosolids by centrifuges. Total coliforms, fecal coliforms, and *E. coli* numbers by plate count	#:<br>(Lau et al., 2017)

				sulI	copies/g	$10^{7.4}$	on mEndo-LES medium, mFC medium, and MFC-BCIG medium respectively. Gene copy numbers from qPCR. Numbers per gram dry weight of biosolids. Average numbers from n = 3 samples for bacteria count and n = 4 samples for gene numbers. Temperature during anaerobic digestion unspecified.<br><br>Biosolids used as fertilizer on agricultural land and for remediation of mine tailing ponds from mining industry. Landfill as contingency option.	PT&TP: (Lau et al., 2017; Region of Waterloo, 2018)
				strA	copies/g	$10^{6.7}$		
	Total coliforms	CFU/g	$10^{6.6}$	*strB*	copies/g	$10^{5.6}$		
	Fecal coliforms	CFU/g	$10^{6.0}$					
	E. coli	CFU/g	$10^{5.8}$					
**Aerobic digestion**								
Campo Real, Madrid, Spain (cake 14.2% solids)<br><br>**	Total	CFU/g	$10^{11.2}$				Activated sludge treatment, biosolids from unknown sludge mix. Gravity thickening of the sludge before digestion. Biosolids are air-dried after digestion. Bacteria numbers by heterotrophic plate count on R2A media with antifungal cyclohexamide (200 ug/ml), fecal coliforms numbers by plate count on TTC medium. Numbers per gram dry weight of biosolids, average numbers from unknown number of samples. Temperature during aerobic digestion unspecified.<br><br>Biosolids used as fertilizer on agricultural land.	#: (Gondim-Porto et al., 2016)<br><br>PT&TP: (Gondim-Porto et al., 2016; Canal de Isabel II S.A., 2017)
	amp resistant (32 ug/mL Amp)	CFU/g	$10^{9.1}$					
	Fecal coliforms	CFU/g	$10^{6.8}$					
Tillsonburg, Ontario, Canada (slurry 1.6% solids)<br><br>***				Total (rrnS)	copies/g	$10^{10.2}$	Activated sludge treatment, biosolids from unknown sludge mix. Aerobic digestion. Total coliforms, fecal coliforms, and *E. coli* numbers by plate count on mEndo-LES medium, mFC medium, and MFC-BCIG medium respectively. Gene copy numbers from qPCR. Numbers per gram dry weight of biosolids. Average numbers from n = 3 samples for bacteria count and n = 4 samples for gene numbers. Temperature during aerobic digestion unspecified.<br><br>Biosolids used as fertilizer on agricultural land.	#: (Lau et al., 2017)<br><br>PT&TP: (Oxford County; Lau et al., 2017; Oxford Country, 2017)
				sulI	copies/g	$10^{10.6}$		
				strA	copies/g	$10^{8.9}$		
				strB	copies/g	$10^{7.8}$		
	Total coliforms	CFU/g	$10^{6.6}$					
	Fecal coliforms	CFU/g	$10^{5.4}$					
	E. coli	CFU/g	§ $< 10^{3.6}$					
Tillsonburg, Ontario, Canada (cake 17.7 % solids)<br><br>***				Total (rrnS)	copies/g	$10^{8.8}$	Activated sludge treatment, biosolids from unknown sludge mix. Aerobic digestion followed by dewatering by centrifuges. Total coliforms, fecal coliforms, and *E. coli* numbers by plate count on mEndo-LES medium, mFC medium, and MFC-BCIG medium respectively. Gene copy	#: (Lau et al., 2017)<br>PT&TP: (Oxford County; Lau et al., 2017;
				sulI	copies/g	$10^{9.3}$		
				strA	copies/g	$10^{7.9}$		
	Total coliforms	CFU/g	$10^{6.7}$	*strB*	copies/g	$10^{6.7}$		

	Fecal coliforms CFU/g $10^{6.3}$<br>*E. coli* CFU/g $10^{6.2}$		numbers from qPCR. Numbers per gram dry weight of biosolids. Average numbers from n = 3 samples for bacteria count and n = 4 samples for gene numbers.<br>Temperature during aerobic digestion unspecified.<br><br>Biosolids used as fertilizer on agricultural land.	Oxford Country, 2017)
**Lime stabilization**				
Lansing, Michigan, USA<br>Lime stabilization only<br>(slurry 9.2% solids)<br>(pH unknown)<br><br>*	Total CFU/g $10^{9.0} – 10^{9.4}$<br><br>*tet* resistant CFU/g $10^{5.4} – 10^{5.6}$<br>(16 ug/mL Tet)<br><br>*sul* resistant CFU/g $10^{7.2} – 10^{8.0}$<br>(50.4 ug/ml Sul)	Total (16S rRNA) copies/g $10^{11.5} – 10^{12.3}$<br><br>*tetW* copies/g $10^{7.3} – 10^{8.3}$<br>*tetO* copies/g $10^{7.4} – 10^{7.6}$<br><br>*sulI* copies/g $10^{6.8} – 10^{7.0}$	Activated sludge treatment, biosolids from unknown sludge mix. Bacteria numbers by heterotrophic plate count on R2A media with antifungal cyclohexamide (200 ug/ml), gene copy numbers from qPCR, numbers per gram of biosolids (wet or dry weight not specified), absolute ranges from n = 2 samples taken at different times of the year.<br><br>Biosolids used as fertilizer on agricultural land.	#:<br>(Munir et al., 2011)<br><br>PT&TP:<br>(Munir et al., 2011)
**Advanced lime stabilization**				
St. Marys, Ontario, Canada<br>Lystek proprietary process alkali + heat + high shear mixing<br>(LysteGro)<br>(slurry 10.8% soilds)<br>(pH 7.9 in final product)	Total coliforms CFU/g $10^{4.1}$<br>Fecal coliforms CFU/g $10^{3.8}$<br>*E. coli* CFU/g §§ $< 10^{2.3}$	Total (rrnS) copies/g $10^{8.4}$<br><br>*sulI* copies/g $10^{8.0}$<br><br>*strA* copies/g $10^{8.2}$<br>*strB* copies/g $10^{6.8}$	Activated sludge anoxic/oxic biological treatment. Biosolids from gravity thickened wasted activated sludge. The thickened sludge is dosed with polymer and dewatered by centrifuges. Dewatered sludge is run through the proprietary Lystek process which involves a combination of heat, alkali, and high shear mixing. Total coliforms, fecal coliforms, and *E. coli* numbers by plate count on mEndo-LES medium, mFC medium, and MFC-BCIG medium respectively. Gene copy numbers from qPCR. Numbers per gram dry weight of biosolids. Average numbers from n = 3 samples for bacteria count and n = 3 samples for gene numbers.<br><br>Biosolids marketed as LysteGro and used as fertilizer on agricultural land.	#:<br>(Murray et al., 2019)<br><br>PT&TP:<br>(Lystek International; Hornick and Blake, 2019)
Thorold, Ontario, Canada<br>Anaerobic digestion + lime stabilization + heat drying<br>(N-Viro/N-Rich)		Total (rrnS) copies/g $10^{5.2}$<br><br>*sulI* copies/g §§ $< \approx 10^{4}$	Niagara Biosolids Processing Facility in Thorold, Ontario, which receives dewatered anaerobic digested sludge (typically 30% solids) from treatment plants in the Niagara Region and from the City of Toronto. The biosolids are mixed with cement kiln dust (primarily calcium oxide), byproduct	#:<br>(Murray et al., 2019)<br>PT&TP:

(dry granulate 59.3% soilds)<br>(pH 11.3 in final product)	Total coliforms<br>Fecal coliforms<br>*E. coli*	CFU/g<br>CFU/g<br>CFU/g	§§<br>§§<br>§§	< $10^{2.3}$<br>< $10^{2.3}$<br>< $10^{2.3}$	*strA*<br>*strB*	copies/g<br>copies/g	§§<br>§§	< ≈10^{4}<br>< ≈10^{4}	from a nearby cement plant. The reaction is exothermic and raises the temperature (temperature unspecified), and it also raises the pH to about 12. This mix is then dried in a direct-fired rotary dryer system for 10 min (temperature unspecified). Plant operated by Walker Industries and uses their N-Viro process. Total coliforms, fecal coliforms, and *E. coli* numbers by plate count on mEndo-LES medium, mFC medium, and MFC-BCIG medium respectively. Gene copy numbers from qPCR. Numbers per gram dry weight of biosolids. Average numbers from n = 3 samples for bacteria count and n = 3 samples for gene numbers.<br><br>Biosolids marketed as N-Rich and used as fertilizer on agricultural land.	(Gunn, 2015; Walker Industries, 2017)
**Heat drying**										
Windsor, Ontario, Canada<br>(dry pellets 95.3 % solids)<br>(2014)<br><br>****<br>*5	Total coliforms<br>Fecal coliforms<br>*E. coli*	CFU/g<br>CFU/g<br>CFU/g	§§<br>§§<br>§§	< $10^{2.3}$<br>< $10^{2.3}$<br>< $10^{2.3}$	Total (rrnS)<br>*sulI*<br>*strA*<br>*strB*	copies/g<br>copies/g<br>copies/g<br>copies/g		$10^{9.1}$<br>$10^{8.4}$<br>$10^{7.4}$<br>$10^{6.3}$	Pellets produced at Windsor Biosolids Processing Facility, which receives dewatered and centrifuged biosolids (approx. 30% solids) from two WWTPs: Little River Pollution Control Plant and Lou Romano Water Reclamation Plant. The two WWTPs uses activated sludge treatment, with biological aerated filters at Lou Romano and aeration tanks at Little River. The biosolids are from a mix of primary and activated sludge. The biosolids are further heat dried and pelletized at the Windsor Biosolids Processing Facility. Total coliforms, fecal coliforms, and *E. coli* numbers by plate count on mEndo-LES medium, mFC medium, and MFC-BCIG medium respectively. Gene copy numbers from qPCR. Numbers per gram dry weight of biosolids. Average numbers from n = 3 samples for bacteria count and n = 4 samples for gene numbers.<br>Temperature during heat drying 400 – 450 °C with a typical retention time of 20 min.<br><br>Biosolids used as fertilizer on agricultural land.	#:<br>(Lau et al., 2017)<br>PT&TP:<br>(City of Windsor; Lau et al., 2017)
Windsor, Ontario, Canada<br>(dry pellets 93.2% solids)<br>(2017)					Total (rrnS)<br>*sulI*	copies/g<br>copies/g		$10^{8.6}$<br>$10^{8.1}$	Pellets produced at Windsor Biosolids Processing Facility, which receives dewatered and centrifuged biosolids (approx. 30% solids) from two WWTPs: Little River Pollution Control Plant and Lou Romano Water Reclamation Plant. The two	#:<br>(Murray et al., 2019)

*5	Total coliforms CFU/g $10^{3.8}$<br>Fecal coliforms CFU/g $10^{3.7}$<br>*E. coli* CFU/g §§ $< 10^{2.3}$	*strA* copies/g $10^{7.8}$<br>*strB* copies/g $10^{6.4}$	WWTPs uses activated sludge treatment, with biological aerated filters at Lou Romano and aeration tanks at Little River. The biosolids are from a mix of primary and activated sludge. The biosolids are further heat dried and pelletized at the Windsor Biosolids Processing Facility. Total coliforms, fecal coliforms, and *E. coli* numbers by plate count on mEndo-LES medium, mFC medium, and MFC-BCIG medium respectively. Gene copy numbers from qPCR. Numbers per gram dry weight of biosolids. Average numbers from n = 3 samples for bacteria count and n = 3 samples for gene numbers.<br>Temperature during heat drying 400 – 450 °C with a typical retention time of 20 min.<br><br>Biosolids used as fertilizer on agricultural land.	PT&TP:<br>(City of Windsor; Murray et al., 2019)
Milwaukee, Wisconsin, USA<br>(dry granulate >90% solids)		Total (16S rRNA) copies/g $10^{11.0}$<br>*tetL* copies/g $10^{6.0}$<br>*tetO* copies/g $10^{7.0}$<br>*sulI* copies/g $10^{8.0}$	Heat-dried biosolids from a blend of wasted activated sludge and anaerobically digested primary sludge from an unspecified municipal WWTP. Gene copy numbers from qPCR. Numbers are reported per gram dry weight.<br>Temperature during heat drying unspecified.<br><br>Use of biosolids unspecified.	#:<br>(Kimbell et al., 2018)<br>PT&TP:<br>(Kimbell et al., 2018)
**Heat drying and pyrolysis**				
Milwaukee, Wisconsin, USA<br>(dry biochar)<br>(300 °C for 60 min)		Total (16S rRNA) copies/g $10^{6.0}$<br>*tetL* copies/g §§ $< 10^{3.9}$<br>*tetO* copies/g §§ $< 10^{2.9}$<br>*sulI* copies/g §§ $< 10^{3.9}$	Lab scale experiment. Pyrolysis of already heat-dried biosolids. The heat-dried biosolids are from a blend of wasted activated sludge and anaerobically digested primary sludge from an unspecified municipal WWTP. Gene copy numbers from qPCR. Numbers are reported per gram dry weight of the biochar remains after pyrolysis.	#:<br>(Kimbell et al., 2018)<br><br>PT&TP:<br>(Kimbell et al., 2018)
Milwaukee, Wisconsin, USA<br>(dry biochar)<br>(500 °C for 5 min)		Total (16S rRNA) copies/g $10^{7.2}$<br>*tetL* copies/g §§ $< 10^{3.9}$<br>*tetO* copies/g §§ $< 10^{2.9}$	Lab scale experiment. Pyrolysis of already heat-dried biosolids. The heat-dried biosolids are from a blend of wasted activated sludge and anaerobically digested primary sludge from an unspecified municipal WWTP. Gene copy numbers	#:<br>(Kimbell et al., 2018)

				sulI	copies/g	§§ < $10^{3.9}$	from qPCR. Numbers are reported per gram dry weight of the biochar remains after pyrolysis.	PT&TP:<br>(Kimbell et al., 2018)
**Soil (for comparison)**								
Nebraska, USA<br>Ungrazed native prairie soil<br><br>*6	Total<br>*tet* resistant<br>(16 ug/mL Tet)<br>Total coliforms<br>*E. coli*	CFU/g<br>CFU/g<br><br>CFU/g<br>CFU/g	$10^{5.0}$ – $10^{5.9}$<br>$10^{4.1}$ – $10^{5.6}$<br><br>$10^{2.3}$ – $10^{4.2}$<br>< 1	*tetA*<br>*sulI*	copies/g<br>copies/g	$10^{4.2}$ – $10^{5.3}$<br>$10^{3.0}$ – $10^{3.4}$	Soil from native prairies where landowners could confirm no grazing by food animals for the last 20 years. Total and resistant bacteria numbers by heterotrophic plate count on R2A media. Coliforms and *E.coli* enumerated using Quantitray (IDEXX Laboratories). Gene copy numbers from qPCR. Numbers per gram dry weight. 20 different sites/prairies studied. The ranges given are the minimum to maximum of the average numbers for each site. 99 of 100 samples had *E. coli* levels lower than 1 CFU/g.	#:<br>(Durso et al., 2016)
Tartu, Estonia<br>"Eerika" experimental station<br>Estonian University of Life Science				Total<br>(16S rRNA)<br>*tetA*<br>*sulI*	copies/g<br><br>copies/g<br>copies/g	$10^{8.0}$ – $10^{8.2}$<br><br>$10^{4.8}$ – $10^{5.1}$<br>< $10^{3.0}$	Experimental field plots with grass. No fertilizer applied for 4 years before the experiment; before that sporadic application of mineral fertilizer. Gene copy numbers from qPCR. Numbers per gram dry weight of soil. 25-75% ranges from 33 soil samples over a time period 151 days.	#:<br>(Nõlvak et al., 2016)
Arganda del Rey, Spain<br>"La Isla" experimental farm	Total<br>*amp* resistant<br>(32 ug/mL Amp)<br>Fecal coliforms	CFU/g<br>CFU/g<br><br>CFU/g	$10^{9.0}$<br>$10^{8.4}$<br><br>$10^{4.4}$				Experimental field plots. No information about history of previous applications of manure, biosolids, or artificial fertilizers. Bacteria numbers by heterotrophic plate count on R2A media with antifungal cyclohexamide (200 ug/ml), fecal coliforms numbers by plate count on TTC medium. Numbers per gram dry weight of soil, average numbers from unknown number of samples.	#:<br>(Gondim-Porto et al., 2016)
London, Ontario, Canada<br>Environmental Science Western Field Station	Total coliforms<br><br>Fecal coliforms	CFU/g<br><br>CFU/g	(n=23) $10^{3.0}$<br>§ (n=1) < $10^{1.9}$<br>(n=10) $10^{2.6}$<br>§ (n=14) < $10^{1.9}$	Total<br>(rrnS)<br>*sulI*<br><br>*strA*<br><br>*strB*	copies/g<br><br>copies/g<br><br>copies/g<br><br>copies/g	$10^{8.6}$<br><br>(n=9) $10^{5.7}$<br>§§ (n=15) < $10^{3.9}$<br>(n=2) $10^{4.1}$<br>§§ (n=22) < $10^{3.9}$<br>(n=1) $10^{4.2}$<br>§§ (n=23) < $10^{3.9}$	Experimental field plots previously cropped with mixed grains and with no known history of manure or biosolids applications. Total and fecal coliforms numbers by plate count on mEndo-LES medium and mFC medium, respectively. Gene copy numbers from qPCR. Numbers are per gram wet weight of soil and the average from 3 fields with different crop types (lettuce, radish, and carrot) each sampled at different times throughout the growth season, 8 samples per field. Total of n = 24 samples.<br>In the measurement of total and fecal coliforms 1 and 14 samples were below the quantification limit of $10^{1.9}$ CFU/(g wet soil) (20 CFU/plate).	#:<br>(Lau et al., 2017)<br>(Tabs. S12 and S14)

		In the measurement of resistance genes 15 samples (Sul-I), 22 samples (Str-A), and 23 samples (Str-B) were below the detection limit of $10^{3.9}$ copies/(g wet soil).

The table includes levels of resistant bacteria and resistance genes reported in the literature for resistance against four selected antibiotics: *ampicillin (amp), tetracycline (tet), sulfonamide (sul), and streptomycin (str).* In addition to the numbers reported from the scientific literature (references marked with #), we have also gathered extended information on plant type, treatment processes, sludge sources, and the normal use of biosolids from each plant from public reports and webpages from municipalities and plant operators (references marked with PT&TP). We have designated sludge/biosolids with solids content below 12% as slurry, sludge/biosolids with solids content from 12-55% as cake, and biosolids with solids content above 55% as dry.

Note that the more advanced methods are not used instead of the simple methods listed at the top of the table, but in addition. E.g., thickened/dewatered sludge is used as input for aerobic or anaerobic digestion, and heat drying is done with sludge that has already been digested and/or thickened/dewatered.

Numbers originally reported as ranges are given as ranges with a note detailing whether the range is absolute (min-max), quartiles, or in any other format. Numbers given as averages are reported as a single average number, and variance numbers are disregarded as there is inconsistency on how these are reported (e.g., as variance, as standard deviation (SD), or even as standard error of the mean (SEM)) and from how many samples they are calculated. All numbers are rounded to one decimal digit in the exponent. All gene numbers are total, most studies do not distinguish between cellular DNA and external DNA.

* These plants showed a significant reduction of resistance genes (copies/100ml) and a significant reduction of resistant bacteria (CFU/100ml) from the raw influent sewage to the treated water effluent, but no significant reduction of resistance genes nor resistant bacteria from the raw influent sewage to the sludge biosolids (Munir et al., 2011).

** It is unknown whether the sludge is aerobically digested in a digestor, or if the aerobic digestion happens during or as part of an air-drying process (the sludge is aerobically digested according to (Gondim-Porto et al., 2016), but no specific information about an aerobic digestor is found on the plant fact sheet from the operator (Canal de Isabel II S.A., 2017)).

*** Note that the gene copy numbers from this study indicate that the aerobic biosolids contains a roughly 3 times higher number of sul1 genes than rrnS genes which were used as a reference for total amount of bacteria. The authors of the study do not comment on this anomality. However, in their Supplementary Material they report that 0.0018 copies of sul1 genes was detected for each copy of the 16S rRNA gene (reference for total amount of bacteria) in a sample of aerobic slurry analyzed by a different method (next generation sequencing) (Lau et al., 2017).

**** The density of living bacteria in the pelletized biosolids from Windsor are below the detection limits in this study, but the number of genes still seems relatively high. However, the same study reports that the abundance of the same gene targets quantified in agricultural soil directly (2-hours, + 24-48 hours storage at 4° C) after application of the pelletized material was remarkably low compared to digested slurry or cake biosolids from other plants. The authors hypothesize that the DNA are carried in non-viable cells or as free DNA segments with lower than normal stability (due to the heat treatment) in the pellets and that it is rapidly destroyed following contact with soil (Lau et al., 2017).

*5 The biosolids from the Windsor Biosolids Pelletizing Facility was tested in both 2014 (Lau et al., 2017) and 2017 (Murray et al., 2019). The plant was built in 1999 and operated from 1999-2019 by American Water (formerly Prism-Berlie) (City of Windsor, 2020). The treatments and processing involved to create the pelletized biosolids have to the authors' knowledge not changed from 2014 to 2017 (Lau et al., 2017; Murray et al., 2019; City of Windsor, 2020).

*6 The Tet-A range is from 18 of 20 of the different prairies, *tetA* was not detected in 2 of the prairies.

§ Numbers below quantification limit, quantification limit given in the respective table cell.

§§ Numbers below detection limit, detection limit given in the respective table cell.

# References for numbers of bacteria and genes.

PT&TP References for plant type and treatment processes.

References

Canal de Isabel II S.A. (2017). Factsheet on EDAR Guadarrama Medio. https://www.canaldeisabelsegunda.es/documents/2014. Available at: https://www.canaldeisabelsegunda.es/documents/20143/638545/Guadarrama+Medio.pdf/39a0572c-b1d1-b4a1-d934-72fcdf155f7a [Accessed September 27, 2021].

City of East Lansing Webpages (2009). City of East Lansing Wastewater Treatment Plant Flow Diagram. Available at: https://www.cityofeastlansing.com/248/Water-Resource-Recovery-Facility [Accessed November 2, 2020].

City of Windsor Lou Romano Water Reclamation Plant. Available at: https://www.citywindsor.ca/residents/environment/Pollution-Control/Laboratory/Pages/Lou-Romano-Water-Reclamation-Plant.aspx [Accessed December 5, 2020].

City of Windsor (2020). Integrated Site Energy Master Plan Study Report for Wastewater Treatment Plants. *Stantec Consult. Ltd*. Available at: https://www.citywindsor.ca/Lists/Tenders/EOI 114-20, Organic Waste Management and Processing/114-20 Addendum 3 COMPLETE - signed.pdf [Accessed December 5, 2020].

Durso, L. M., Wedin, D. A., Gilley, J. E., Miller, D. N., and Marx, D. B. (2016). Assessment of Selected Antibiotic Resistances in Ungrazed Native Nebraska Prairie Soils. *J. Environ. Qual.* 45, 454–462. doi:10.2134/jeq2015.06.0280.

Gondim-Porto, C., Platero, L., Nadal, I., and Navarro-García, F. (2016). Fate of classical faecal bacterial markers and ampicillin-resistant bacteria in agricultural soils under Mediterranean climate after urban sludge amendment. *Sci. Total Environ.* 565, 200–210. doi:10.1016/j.scitotenv.2016.04.160.

Gunn, E. (2015). A Partnership Creates A Biosolids Facility That Yields A Quality Product. *Treat. Plant Oper. Mag.* Available at: https://www.tpomag.com/editorial/2015/03/a_partnership_creates_a_biosolids_facility_that_yields_a_quality_product [Accessed December 5, 2020].

Hornick, R., and Blake, D. (2019). Town of St. Marys Wastewater Treatment and Collection 2019 Wastewater Summary Report. *St. Marys Ontario Canada, 2019*. Available at: https://www.townofstmarys.com [Accessed December 5, 2020].

Imlay City Webpages (2020). Wastewater Treatment. Available at: https://www.imlaycity.org/government/wastewater-treatment/ [Accessed October 12, 2020].

Kimbell, L. K., Kappell, A. D., and McNamara, P. J. (2018). Effect of pyrolysis on the removal of antibiotic resistance genes and class I integrons from municipal wastewater biosolids. *Environ. Sci. Water Res. Technol.* 4, 1807–1818. doi:10.1039/c8ew00141c.

Lau, C. H. F., Li, B., Zhang, T., Tien, Y. C., Scott, A., Murray, R., et al. (2017). Impact of pre-application treatment on municipal sludge composition, soil dynamics of antibiotic resistance genes, and abundance of antibiotic-resistance genes on vegetables at harvest. *Sci. Total Environ.* 587–588, 214–222. doi:10.1016/j.scitotenv.2017.02.123.

Lystek International Biosolids Processing. Available at: https://web.archive.org/web/20201205154619/https://lystek.com/technology/biosolids-processing/ [Accessed December 5, 2020].

Munir, M., Wong, K., and Xagoraraki, I. (2011). Release of antibiotic resistant bacteria and genes in the effluent and biosolids of five wastewater utilities in Michigan. *Water Res.* 45, 681–693. doi:10.1016/j.watres.2010.08.033.

Murray, R., Tien, Y. C., Scott, A., and Topp, E. (2019). The impact of municipal sewage sludge stabilization processes on the abundance, field persistence, and transmission of antibiotic resistant bacteria and antibiotic resistance genes to vegetables at harvest. *Sci. Total Environ.* 651, 1680–1687. doi:10.1016/j.scitotenv.2018.10.030.

Nõlvak, H., Truu, M., Kanger, K., Tampere, M., Espenberg, M., Loit, E., et al. (2016). Inorganic and organic fertilizers impact the abundance and proportion of antibiotic resistance and integron-integrase genes in agricultural grassland soil. *Sci. Total Environ.* 562, 678–689. doi:10.1016/j.scitotenv.2016.04.035.

Oxford Country (2017). 2017 Annual Wastewater Treatment System Summary Report. Available at: https://www.oxfordcounty.ca/Services-for-You/Water-Wastewater/Wastewater/Annual-reports [Accessed October 15, 2020].

Oxford County 2017 Annual Biosolids Management Report. *2017*. Available at: https://www.oxfordcounty.ca/Services-for-You/Water-Wastewater/Wastewater/Annual-reports, [Accessed October 15, 2020].

Region of Waterloo (2018). Region of Waterloo 2018 Wastewater Treatment Master Plan July 2018. Available at: https://www.regionofwaterloo.ca/en/regional-government/water-and-wastewater.aspx.

Traverse City/Jacobs (2018). Traverse City Regional Wastewater Treatment Plant Brochure 2018, Overview of Equipment and Last Installation/Upgrade, Jacobs report number: BI0403181111DEN. Available at: https://www.traversecitymi.gov/wastewaterplant.asp [Accessed November 2, 2020].

Walker Industries (2017). N-Rich Soil Amendment Safety Data Sheet, revision 001. Available at: https://www.walkerind.com/media/2020/01/N-Rich-SDS.pdf [Accessed December 5, 2020].
